# Comparison of stainless and mild steel welding fumes in generation of reactive oxygen species

**DOI:** 10.1186/1743-8977-7-32

**Published:** 2010-11-03

**Authors:** Stephen S Leonard, Bean T Chen, Samuel G Stone, Diane Schwegler-Berry, Allison J Kenyon, David Frazer, James M Antonini

**Affiliations:** 1 Pathology and Physiology Research Branch, Health Effects Laboratory Division, National Institute for Occupational Safety and Health, Morgantown, WV, USA

## Abstract

**Background:**

Welding fumes consist of a wide range of complex metal oxide particles which can be deposited in all regions of the respiratory tract. The welding aerosol is not homogeneous and is generated mostly from the electrode/wire. Over 390,000 welders were reported in the U.S. in 2008 while over 1 million full-time welders were working worldwide. Many health effects are presently under investigation from exposure to welding fumes. Welding fume pulmonary effects have been associated with bronchitis, metal fume fever, cancer and functional changes in the lung. Our investigation focused on the generation of free radicals and reactive oxygen species from stainless and mild steel welding fumes generated by a gas metal arc robotic welder. An inhalation exposure chamber located at NIOSH was used to collect the welding fume particles.

**Results:**

Our results show that hydroxyl radicals (^.^OH) were generated from reactions with H_2_O_2 _and after exposure to cells. Catalase reduced the generation of **^.^**OH from exposed cells indicating the involvement of H_2_O_2_. The welding fume suspension also showed the ability to cause lipid peroxidation, effect O_2 _consumption, induce H_2_O_2 _generation in cells, and cause DNA damage.

**Conclusion:**

Increase in oxidative damage observed in the cellular exposures correlated well with **^.^**OH generation in size and type of welding fumes, indicating the influence of metal type and transition state on radical production as well as associated damage. Our results demonstrate that both types of welding fumes are able to generate ROS and ROS-related damage over a range of particle sizes; however, the stainless steel fumes consistently showed a significantly higher reactivity and radical generation capacity. The chemical composition of the steel had a significant impact on the ROS generation capacity with the stainless steel containing Cr and Ni causing more damage than the mild steel. Our results suggest that welding fumes may cause acute lung injury. Since type of fume generated, particle size, and elapsed time after generation of the welding exposure are significant factors in radical generation and particle deposition these factors should be considered when developing protective strategies.

## Background

There are approximately 390,000 full-time welders in the United States [1] and an estimated 5 million persons occupationally exposed to welding fumes worldwide. The welding process which joins materials by causing coalescence using a filler material, usually wire, to form a molten pool which then cools bonding the surfaces together. During this process an occupational exposure can occur through inhalation of the fume and particles. Although full-time welders may be easier to track, the profession has a large number of part-time, small shop welders who may also be exposed as well as others working in the vicinity of the welding activities. These part-time and cross workplace exposures make the effects difficult to monitor. Welding is frequently carried out in areas with poor ventilation such as ship hulls, metal tanks, or pipe and crawl spaces leading to a greater potential for exposure. Welding fumes have been demonstrated to cause toxicity among exposed workers [2,3] Welding fumes consist of a wide range of metal oxide particles, including iron, manganese, chromium, and nickel, which are generated mostly from the electrode/wire feed [4]. Two of the major feed wire types which are used in the welding process are mild steel (MS) and stainless steel (SS).

Inhalation of the fume has been related to bronchitis [5], metal fume fever, occupational asthma [6] cancer and possible increases in lung tumorigenicity [7,8], suppression of lung defenses [9,10], and functional changes in the lung [11-14]. Investigations have also shown an increase in ROS production after welding fume generation [15-17] and initiation of downstream mediators, such as HO-1, VEGF, and MAP kinases [18-20]. The generated fume ranges in size and can be deposited throughout the respiratory tract [4,21-23]. Previous studies have demonstrated the impact of particle size and surface area on pulmonary effects of inhaled toxicants [24].

**Figure 1:**
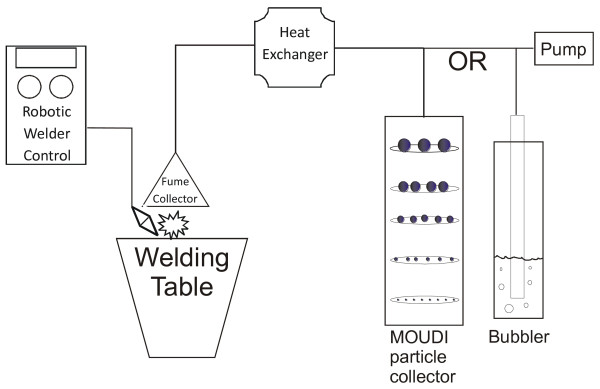
**Schematic of the welding fume generation system**. Welder operated from screened computer control room as welding occurs on a table. Generated fume collected immediately off the surface and passed through a heat exchanger then collected in the MOUDI particle collector or midget impinger.

The effects seen from exposure to welding fume are under investigation but not well understood nor are the mechanisms behind potential toxicity from fume exposure. However, the act of welding causes the generation of unstable metal oxides due to the energy at the point of the weld leading to an uncommon form of first exposure to newly formed unstable and potentially more reactive particles, similar to that seen in sandblasting [16,25-27]. Biological effects of welding fume exposure have been investigated employing cellular [28,29] and animal models [30] using a welding exposure system located at NIOSH Health Effects Laboratory Division. This system can simulate real workplace exposures and allows the collection of fresh welding fume from a continuous weld (figure 1). We used this automated system to collect welding fume from both SS wire and MS wire while using an arc welding process similar to that reported previously [22]. Differences in toxicity between mild steel and stainless steel fume have been previously observed [31,32]. Although, the reasons for these differences are not fully understood, some studies have indicated metal content in the weld wire as being the cause [33-35].

The goal of our study was to access differences in reactive oxygen species (ROS) generation during the welding process. This study will investigate the effects of weld wire type, particle size, surface area and time after generation of fume on the potential generation of ROS and its downstream effects. The observations reported here hope to elucidate the mechanism behind some of the biological effects observed in occupational exposures to welding fume.

## Results

### Particle morphology and characteristics

**Figure 2:**
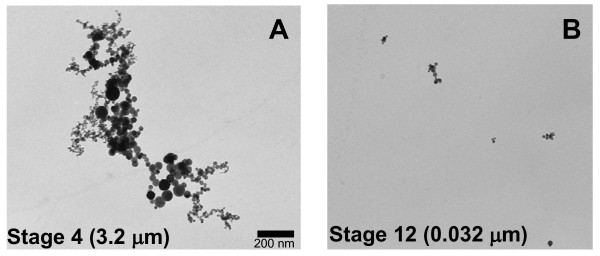
**Electron micrographs of two of the 15 filters used in the MOUDI and nano-MOUDI**. A) shows stage 4 (3.2 μm cut-off) larger particles and the formation of agglomerated chains of particles. B) shows stage 12 (0.032 μm cut-off) smaller individual particles and less chain formation.

Electron micrographs of welding fume sample collected on MOUDI filters (Figure 2) showed large agglomerated chains of particles linked together which not only formed larger aggregate particles but greatly increased surface area. Particle sizes are not related to one single large particle but many smaller spherical particles linked as a chain which by its form creates a much greater surface area than would ordinarily be associated with a single spherical particle of this size. This greater surface area allows much more reaction surface with the generated fumes.

**Figure 3:**
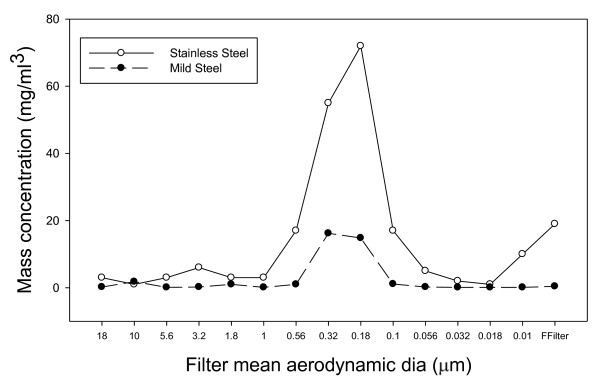
**Mass concentration trapped on each filter of the MOUDI**.

Most particles, calculated by mass, generated in our welding fume system were between 0.56 and 0.1 μm in mean diameter as demonstrated in figure 3. This size range deposits mostly in the alveolar and bronchiolar regions in the lung; however, particles were found at all size ranges measured using the MOUDI. Note that even though grease was not used on the MOUDI stages in this study, the profile of the mass collected on the stages was similar to that obtained when the grease was used [22], demonstrating that the phenomenon of potential particle bounce, if any, was minimal.

Table 1 shows the elemental analysis of both stainless and mild steel wire which indicated that each wire contained Fe, Mn and Cu. However, stainless steel also contained the transition metals Cr (20.2 ± 1.52% bw) and Ni (8.76 ± 0.18% bw) while mild steel showed a small amount of Si (2.75 ± 0.28% bw)

**Table 1:** Stainless steel versus mild steel elemental analysis.

Stainless Steel		
**Metal**	**μg/sample**	**Weight % of metal**

Fe	1207 ± 161	57 ± 1.28

Cr	427.5 ± 69.1	20.2 ± 1.52

Mn	295.0 ± 48.4	13.8 ± 0.45

Ni	185 ± 24.0	8.76 ± 0.18

Cu	3.30 ± 0.492	0.155 ± 0.004

**Mild Steel**		

Fe	776 ± 9.8	80.6 ± 0.17

Mn	142 ± 2.0	14.7 ± 0.11

Si	26.6 ± 2.9	2.75 ± 0.28

Cu	17.2 ± 0.20	1.79 ± 0.02

### Free radical generation and ROS

Free racial generation was examined using three separate systems; sized fume reaction with hydroxyl radical precursor H_2_O_2_, direct bubbling of whole fume through solution containing H_2_O_2 _and the spin trap DMPO, and sized fume effects on exposed cells.

**Figure 4:**
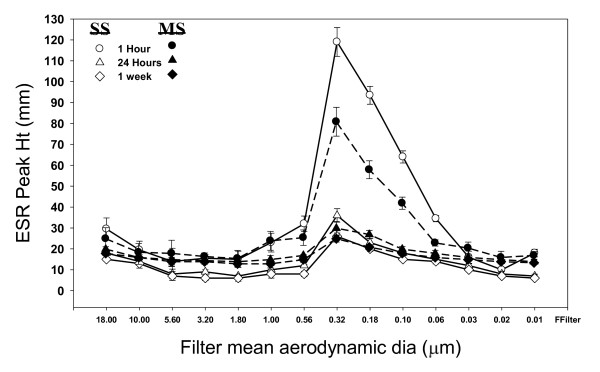
**The generation of short-lived ^.^OH radicals upon reaction of H_2_O_2 _with individual filter sizes using different wire type and time periods after generation of fume**. Open symbols represent stainless steel while filled symbols are mild steel wire. ESR spectrum recorded 3 min after reaction was initiated in PBS (pH 7.4), 1 mM H_2_O_2 _and 100 mM DMPO. ESR settings were; center field, 3385 G; scan width, 100 G; time constant, 40 msec; scans, 5; modulation amplitude, 1 G; receiver gain, 2.5 × 10^4^; frequency, 9.793 GHz; and power, 63 mW.

**Figure 5:**
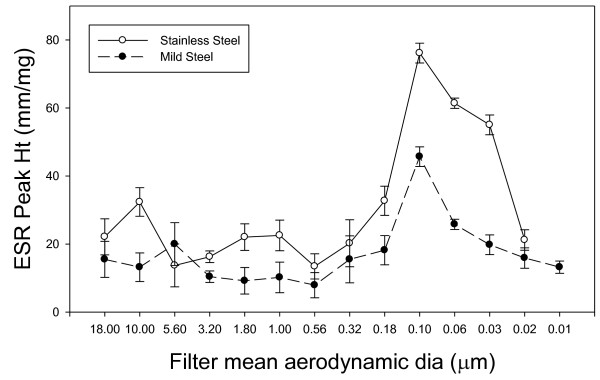
**Mass normalized radical activity, ^.^OH radical peak heights of individual filters sizes adjusted for radicals/mg on 1 hour post generation samples**. ESR settings were; center field, 3385 G; scan width, 100 G; time constant, 40 msec; scans, 5; modulation amplitude, 1 G; receiver gain, 2.5 × 10^4^; frequency, 9.793 GHz; and power, 63 mW. Asterisks indicate a significant increase in stainless steel fume compared to mild steel fume (P < 0.05)

Figure 4 shows individual filter sized welding fume reacting with hydrogen peroxide to measure generation of hydroxyl radical (**^.^**OH). Strong radical generation is observed at the 1 hour post collection time in both the stainless steel and mild steel samples. The number of radicals generated is reduced over time as shown at the 24 hour and 1 week post generation. Using the 0.32 μm mean aerodynamic diameter as a reference; stainless steel showed a 68% reduction in **^.^**OH radical signal between1 hour to 24 hours post generation which became a 77% reduction at the 1 week time point. Mild steel showed similar reduction over time with a 64% drop in **^.^**OH signal from 1 hour to 24 hours and a 67% drop at the 1 week time point. The strongest **^.^**OH radical signals were measured at the 0.32 μm to 0.056 μm size ranges; however, it should be noted this closely corresponds to the observed higher mass collected at those sizes. When the **^.^**OH radical signal was adjusted for particle weight/filter, mass normalized radical activity, it was observed that the smaller particles had increased radical generation potential per unit weight, figure 5. Stainless steel showed higher generation of **^.^**OH radicals at every time point (except 5.6 μm) with significantly higher generation; 41%, 57% and 59% at sizes 0.1 μm, 0.056 μm, and 0.032 μm respectively. Addition of deferoxamine, a metal chelator showed a decrease in radical signal strength in a concentration dependent manner with a complete abolishment of the DMPO/**^.^**OH signal at 2 mM deferoxamine. Additon of catalase, an H_2_O_2 _catalyst, to the cellular reactions decreased the radical signal as well (data not shown).

**Figure 6:**
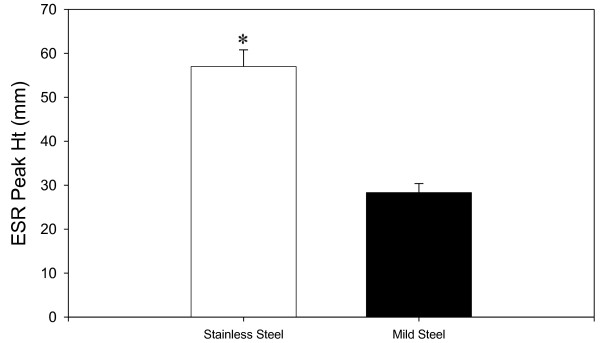
**^.^OH radical peak heights of midget impinger bubbled samples**. Flow rate 3.80 L/min, 20 min collection time. Welding fume was bubbled directly through PBS (pH 7.4), 1 mM H_2_O_2 _and 100 mM DMPO. ESR settings were; center field, 3385 G; scan width, 100 G; time constant, 40 msec; scans, 1; modulation amplitude, 1 G; receiver gain, 2.5 × 10^4^; frequency, 9.793 GHz; and power, 63 mW. Asterisks indicate a significant increase in stainless steel fume compared to mild steel fume (P < 0.05)

Figure 6 shows the results of total fumes drawn directly from the welding surface and bubbled through PBS in the presence of H_2_O_2 _and DMPO, to act as a spin trap. Results demonstrate the total sample potential to generate ^.^OH radicals when reacted with H_2_O_2_. Stainless steel fumes showed a significantly higher generation of ^.^OH radicals than was an equal mass of fume generated by mild steel.

**Figure 7:**
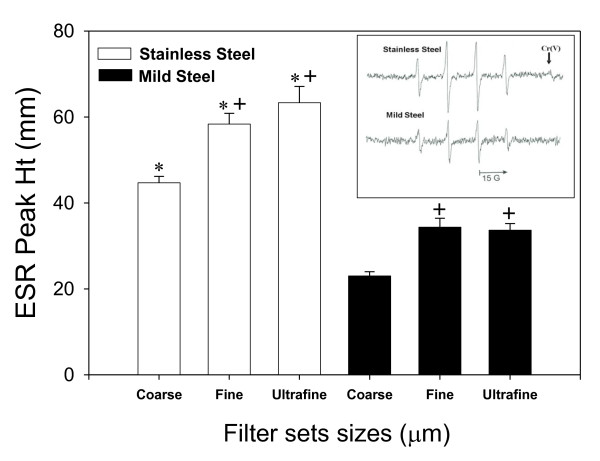
**Radicals generated from RAW 264.7 cells (1 × 10^6^/ml) exposed to grouped filter sample welding fume (250 μg/ml) for 10 min at 37°C in a shaking water bath**. ESR settings were; center field, 3385 G; scan width, 100 G; time constant, 40 msec; scans, 5; modulation amplitude, 1 G; receiver gain, 2.5 × 10^4^; frequency, 9.793 GHz; and power, 63 mW. Asterisks indicate a significant increase in radicals compared between steel types; (+) indicate significant difference between fume sizes.

The results of RAW 264.7 cellular exposure to welding fume by size group and type of fume are shown in figure 7. Stainless steel fumes incubated with cells showed a significant increase at all three size groups when compared to mild steel fumes. There was also an increase in radical production within each steel type as particle size decreased. It should also be noted that the Cr (V) electron spin resonance signal (figure 7 inset) was observed only in spectra from stainless steel fume exposure to cells.

### Lipid peroxidation

**Figure 8:**
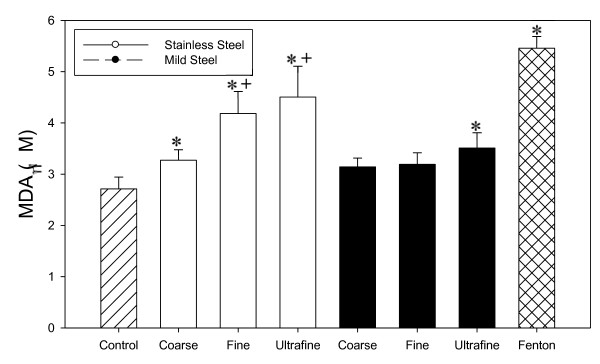
**Welding fume induced lipid peroxidation in incubation mixture containing 250 μg/ml welding fume sample and 5 × 10^7 ^RAW 264.7 cells**. Data presented are means of ± S.D. for 4 sets of experiments. (*) indicate a significant increase in lipid peroxidation compared to control. (+) indicate significant difference between metal types at the same sizes. (P < 0.05)

Stainless steel fumes showed a significant increase in lipid peroxidation in exposed RAW 264.7 cells compared to control at all three sizes groups at equal mass as seen in figure 8. Mild steel fumes caused a significant increase at the ultrafine particle size group. Stainless steel also showed a significant increase over mild steel when comparing fine and ultrafine size groups.

### H_2_O_2 _production

**Figure 9:**
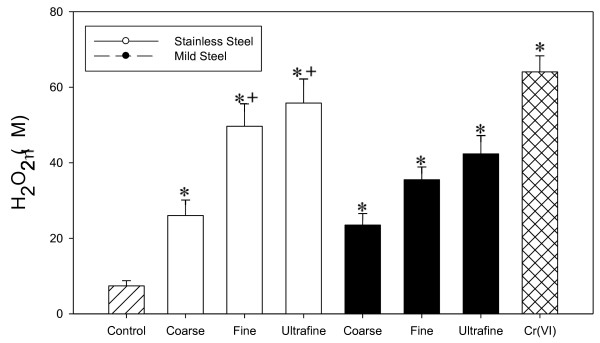
**H_2_O_2 _production in incubation mixtures containing 250 μg/ml welding fume sample and 1 × 10^6 ^RAW 264.7 cells**. Data presented are means of ± S.D. for 4 sets of experiments. (*) indicate a significant increase in lipid peroxidation compared to control. (+) indicate significant difference between metal types at the same sizes. (P < 0.05)

Figure 9 shows the effects of welding fume on H_2_O_2 _generation after RAW 264.7 cells were exposure to welding fume. All welding fume sizes and steel types at equal mass showed a significant rise in H_2_O_2 _production in exposed RAW 264.7 cells when compared to control. A significant difference was also observed between stainless steel and mild steel fumes in the fine and ultrafine size groups. A size dependent increase in H_2_O_2 _production was also observed in both types of metals.

### O_2 _consumption

**Figure 10:**
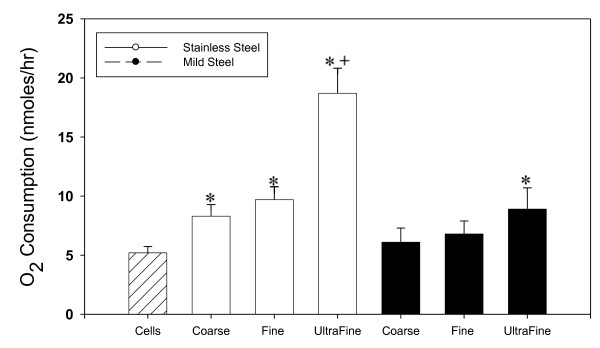
**Oxygen consumption in welding fume stimulated RAW 264.7 cells**. Incubation mixtures contain 3 × 10^6 ^RAW 264.7 cells + 500 μg/ml welding fume sample. Data presented are means of ± S.D. for 4 sets of experiments. (*) indicate a significant increase in oxygen consumption compared to control. (+) indicate significant difference between metal types at the same sizes. (P < 0.05)

A significant rise in oxygen consumption was observed in RAW 264.7 cells in all stainless steel size exposures and in the mild steel ultrafine size as shown in figure 10. A significant difference was observed between stainless steel fumes and mild steel fumes at equal mass in the ultrafine size group. A size dependent increase was also observed within steel types.

### DNA damage

**Figure 11:**
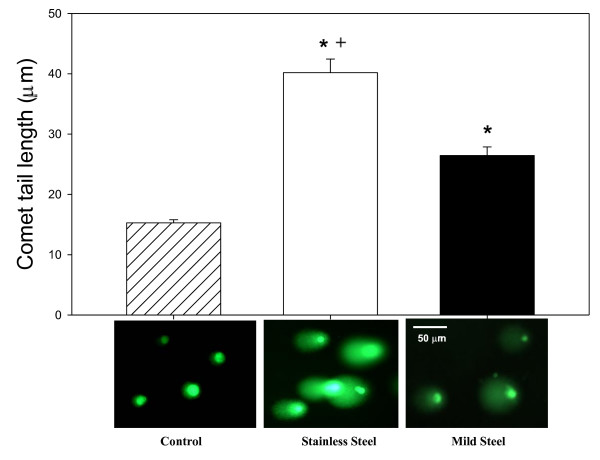
**Comet assay results after incubation of RAW 264.7 cells for 1 h exposed to 250 μg/ml welding fume**. Data presented are means of ± S.D. for 4 sets of experiments. (*) indicate a significant increase in DNA damage compared to control. (+) indicate significant difference between metal types at the same sizes. (P < 0.05)

Comet assay results for DNA damage are shown in figure 11. The data demonstrates total grouped size filters for both stainless and mild steel at equal mass showed a significant increase in DNA damage in exposed RAW 264.7 cells. Furthermore, the stainless steel fumes caused significantly more DNA damage when compared to mild steel fumes.

## Discussion

The present study was undertaken to investigate possible radical generation of fume generated from two of the most common types of welding processes to access potential cellular damage from ROS generation and to determine if the biological effects were size, time and surface area dependent. Our results determined that both stainless steel and mild steel welding fumes are able to generate **^.^**OH radicals in a Fenton-like system. These **^.^**OH radials are precursors and initiators of many forms of ROS that can produce damage to cellular membranes, proteins and DNA as well as initiate further downstream damage and signaling associated with respiratory burst and inflammation. Our results also determined that freshly generated fume samples, 1 hour post, were significantly more reactive in generating **^.^**OH radials than samples aged for 24 hours and 1 week. This reactivity is attributed to the change in transition states of the freshly generated metals that are produced during the welding process due to the high energy involved. Transition metals have the ability to accept and donate single electrons. Metals used in welding may temporarily attain a different and possibly more reactive, transition or valence state and in this changed transition state they are able to overcome the spin restriction on direct reaction of O_2 _with non-radicals. These reactions can lead to the generation of ROS and further damage to biological systems. Addition of metal clelators and catalase confirmed the involvement of metals and H_2_O_2 _in the radical generation observed. A consistent result throughout our various ESR measurements (H_2_O_2_, cellular and direct whole fume) was the ability of stainless steel fumes to generate higher amounts of free radicals than mild steel. Animal studies indicate that stainless steel welding fumes induce more lung inflammation and injury compared to mild steel fumes [17] Elemental analysis of the fumes showed that stainless steel contained two transition metals not found in mild steel, chromium and nickel. Both chromium and nickel have had extensive research performed on their reactivity and abilities to produce ROS in biological systems [36]. These metals have been shown to be highly toxic and result in generation of ROS and associated damage. Furthermore, their ability to cycle through transition states once in a cellular system can lead to extensive and ongoing ROS production. This metal cycling ability was demonstrated in our investigation by the ESR spectra results showing the production of Cr(V) in stainless steel fume exposed cells. This Cr(V) radical signal came from the reduction of Cr(VI) in the welding fume in a cellular system. This Cr(VI) → Cr(V) system has been researched and shown to be toxic and to responsible for the generation ROS associated damage [37].

The relationship between free radical generation and different sizes at equal mass was also investigated to determine the effect on free radical generation after exposure to H_2_O_2_. The greatest free radical generation was observed when analyzing the ultrafine particles demonstrating that reactivity of the particles is dependent on available surface area with equal mass. Generated welding fumes are long chains of agglomerated particles which are trapped on filters in size groups. However, the large chains are actually made up of many smaller primary particles linked together, creating a much higher surface area at equal mass than spherical particles that are more commonly observed in the environment and workplace. The welding fume chains formed under the high heat conditions of welding acts synergistically to create fresh particles of a high surface area which generate reactive species. Freshly generated welding fume has been previously reported to be more biologically active in an animal model [16].

Welding fumes were also found to cause lipid peroxidation, an indicator of cell membrane damage and precursor for other radical generation systems. Lipid peroxidation results in the release of lipid-derived radicals (R**^.^**, RO**^. ^**and ROO**^.^**) [38], which can lead to a cascade effect and release reactive iron [39] causing the generation of more ROS. Lipid peroxidation and its effects have been found to cause DNA damage [40] and may function as tumor initiators [41]. Once again the stainless steel fumes cause significantly greater damage to the cells than mild steel. This same trend, as well as a trend in smaller size i.e. greater surface area was also demonstrated in H_2_O_2 _generation, O_2 _consumption and DNA damage in exposed RAW 264.7 cells. H_2_O_2 _generation and O_2 _consumption are indicators of a respiratory burst in the exposed cells creating downstream effects and activation of inflammatory pathways and signaling. The significantly higher DNA damage observed in the stainless steel fumes may be due to the different metals present. Hydroxyl radicals generated with certain metals, such as nickel and copper, exhibit less reactivity with DNA. Possible reasons for this non-reactivity include; that OH radicals are generated within the domain of certain macromolecules and, therefore, are not able to exhibit significant reactivity and the structural contribution of metal toward DNA-binding and metal interactions with the DNA [42,43]. The results from our study show that OH radicals generated from both welding fumes have the potential to cause DNA damage with stainless steel containing Cr causing significantly greater DNA damage. In addition, H_2_O_2 _and OH generated by cells exposed to welding fume may cause other cellular damage via mechanisms associated with reactions initiated by ROS, for example, dG hydroxylation and protein-DNA cross-links. Furthermore these ROS may also cause activation of nuclear transcription factors, such as NF-κB, over-expression of certain oncogenes and induction of p53 mutation [44,45].

## Conclusions

The significant increase in oxidative damage observed in the cellular exposures correlates well with the results as determined by ESR of **^.^**OH generation in size and type of welding fumes, indicating the influence of metal type and transition state on radical production as well as associated damage. Our results demonstrate that both types of welding fumes are able to generate ROS and ROS-related damage over a range of particle sizes; however, the stainless steel fumes consistently showed a significantly higher reactivity and radical generation capacity. The chemical composition of the steel had a significant impact on the ROS generation capacity with the stainless steel, but not mild steel, containing Cr and Ni causing more damage than the mild steel. Both materials contained Fe, a known Fenton radical producer, Mn and Cu. Our results suggest that the marked difference between the radicals produced in the two types of fumes is due to the presence of Cr found in the stainless steel. Cr has been shown in many investigations to a cause a number of toxicities. It is also noted that the smaller particle size of the fume the greater the ROS potential at equal mass with the 0.180 - 0.032 μm sizes showing the most reactivity in both types of welding fume which can penetrate deepest into the lung of an exposed welder. Our results further demonstrated that the freshly generated fumes were more reactive and caused more oxidative damage than the aged particles, indicating that metal transition state also plays an important role in welding fume reactivity. Therefore, our results suggest that welding fumes may cause acute lung injury. Since type of fume generated, particle size, and elapsed time after generation of the welding exposure are significant factors in radical generation and particle deposition these factors should be considered when developing protective strategies.

## Methods

### Reagents

Chelex 100 resin was purchased from Bio-Rad Laboratories (Richmond, CA, USA). Phosphate-buffered saline (PBS), (KH_2_PO_4 _(1.06 mM), Na_2_HPO_4 _(5.6 mM), NaCl (154 mM), pH 7.4), was purchased from Biowhittaker Inc. (Walkersville, MD, USA). The PBS was treated with Chelex 100 to remove transition metal ion contaminants. Dulbecco's modified eagles medium (DMEM), 5,5-dimethyl-1-pyroline-oxide (DMPO), fetal bovine serum (FBS), FeSO_4, _H_2_O_2, _and penicillin/streptomycin were purchased from Sigma Chemical Company (St. Louis, MO, USA). The spin trap, DMPO, was purified by charcoal decolorization and vacuum distillation and was free of ESR detectable impurities. Quartz sample tubes were purchased from Wilmad Glass (Buena, NJ, USA).

### Cell culture

RAW 264.7 mouse peritoneal monocytes were purchased from American Type Culture Collection (Rockville, MD). RAW 264.7 cells are commonly used and have been found to respond to particle exposure in a manner similar to primary alveolar macrophages [46-49]. RAW 264.7 cells were cultured in DMEM with 10% FBS, 2 mM L-glutamine, and 50 mg/ml pen/strep at 37°C in a 5% CO_2 _incubator. Cells were split after confluence approximately every 3 days.

### Generation and collection of welding fumes

The welding fume generation system was similar to that previously outlined [22]. Briefly, the generation system consisted of a welding power source (Power Wave 455, Lincoln Electric, Cleveland, OH), an automated, programmable six-axis robotic arm (Model 100 Bi, Lincoln Electric), a water cooled arc welding torch (WC 650 amp, Lincoln Electric), a wire feeder that supplied the wire to the torch at a programmed rate up to 300 inches/min, and an automatic welding torch cleaner that kept the welding nozzle free of debris and spatter. Gas metal arc welding was performed using a mild steel electrode (carbon steel ER70S-6, Lincoln Electric) or a stainless steel electrode (Blue Max E308LSi wire, Lincoln Electric). Welding took place on A36 carbon steel plates for fume collection times of 20 min at 25 V and 200 amps. During welding, a shielding gas combination of 95% Ar and 5% CO_2 _(Airgas Co., Morgantown, WV) was continually delivered to the welding nozzle at an air flow of 20 L/min.

Figure 1 shows an outline of the generation and collection system. The system is contained in three rooms; the control room, the robotic arm welding fume generator and the fume collection chamber. All three rooms were separated from each other.

Aerodynamically size-selected aerosol samples were collected with the Micro-Orifice Uniform Deposit Impactor (MOUDI), model # 110 and Nano-MOUDI MSP Model #115, with rotator (MSP, Inc., Minneapolis, MN, USA). This MOUDI/Nano-MOUDI arraignment provided a 15-stage research-grade cascade impactor. Each filter stage has a cutoff size with particles collected at each stage being aerodynamically size-selected between stage sizes. Cutoff sizes were; 18, 10, 5.6,3.2, 1.8, 1.0, 0.56, 0.32, 0.18, 0.10, 0.006, 0.03, 0.02, 0.01 μm and the final filter. Filters used for ESR and cellular exposures were from Millipore Corp. (Billerica, MA, USA) 47 mm, 0.8 μm, PVC model PVC0847600. PVC was selected because it has previously been demonstrated to have no effect in the free radical analysis. The MOUDI substrates are normally coated with grease to ensure adherence of deposited particles and to avoid bounce of large particles to lower stages of the impactor. However, grease can alter the surface of collected aerosol particles and is not suitable for use in collecting samples for free radical analysis. Therefore, the cascade impactor was operated without grease substrates to collect and fractionate the welding fume. Filters were collected for 20 min. Filters were then split into 3 groups for either 1 hour, 24 hour or 1 week post generation analysis.

Filter suspensions, which were used for H_2_O_2_, lipid peroxidation, oxygen consumption and DNA damage analysis, were prepared by splitting the filters into three size groups. For our study the size groups were defined as ultrafine (0.01 - 0.056 μm), fine (0.1 - 1.0 μm) and coarse (1.8 - 18 μm) particles. The groups consisted of five filters each of which were placed in PBS. Pre- and post filter weights were used to prepare a final concentration of 1 mg/ml welding fume suspension. The slurry was centrifuged, and the fume suspension was decanted from the filter pellet. A clean control suspension was also prepared at a ratio of 4 filters/1 ml PBS.

### Welding particle morphology, transmission electron microscopy (TEM)

Welding fume samples, collected separately from the fumes used in free radial analysis, were collected at 30-minute intervals during 3 h of welding directly onto formvar-coated TEM grids and viewed using a JEOL 1220 transmission electron microscope (JOEL, Inc.).

### Welding particle size distribution

Particle size distribution was determined by using a Micro-Orifice Uniform Deposit Impactor (MOUDI) model # 110 which is intended for the general purpose aerosol sampling, and a Nano-MOUDI (MSP Model 115) that is specifically designed for sampling aerosols in the size range down to 0.010 μm.

### Welding particle metal content analysis

Mild and stainless steel welding particles were collected onto 5.0 mm polyvinyl chloride membrane filters in 37 mm cassettes during 30 min of welding. Metal analysis samples were collected separately from the samples used for free radical analysis. The particle samples were digested and the metals analyzed by inductively coupled plasma atomic emission spectroscopy (ICP-AES) by Clayton Group Services (A Bureau Vertis Company, Novi, MI) as coordinated with the Division of Applied Research and Technology (DART, Cincinnati, OH) according to NIOSH method 7300 modified for microwave digestion [50]. Metal content of blank filters also was analyzed for control purposes.

### Free radical measurements

ESR spin trapping was used to detect short-lived free radical intermediates. Hydroxyl radicals were measured using the addition-type reaction of a short-lived radical with a compound (spin trap) to form a relatively long-lived paramagnetic free radical product (spin adduct), which can then be studied using conventional ESR. For hydroxyl radical measurements on individual filter sizes, reactants were mixed in test tubes at a final volume of 1.0 ml of PBS in the presence of 1 mM H_2_O_2_. The reaction mixture was then transferred to a quartz flat cell for ESR measurement. Experiments were performed at room temperature and under ambient air. The concentrations given in the figure legends are final concentrations. The intensity of the signal is used to measure the relative amount of short-lived radicals trapped, and the hyperfine couplings of the spin adduct are characteristic of the original trapped radicals. Spin trapping is the method of choice for detection and identification of free radical generation due to its specificity and sensitivity. All ESR measurements were conducted using a Bruker EMX spectrometer (Bruker Instruments Inc. Billerica, MA 01821, USA) and a flat cell assembly. Hyperfine couplings were measured (to 0.1 G) directly from magnetic field separation using potassium tetraperoxochromate (K_3_CrO_8_) and 1,1-diphenyl-2-picrylhydrazyl (DPPH) as reference standards [51,52]. The relative radical concentration was estimated by multiplying half of the peak height by (ΔH_pp_)^2^, where ΔH_pp _represents peak-to peak width. The Acquisit program was used for data acquisitions and analyses (Bruker Instruments Inc. Billerica, MA 01821, USA).

### Radicals trapped by bubbler

Freshly generated total welding particle reactivity was measured using a midget bubbler (Ace Glass, Vineland NJ) containing a reaction mixture made up of H_2_O_2 _(10 mM) DMPO (100 mM), and PBS to instantly trap generated radicals for ESR measurement. The midget bubbler was attached to a collection tube with a flow rate of 1 L/min positioned approximately 18 inches from the welding surface in order to directly collect the freshly generated fume. Fume was collected for 20 min. The reaction mixture was then immediately measured using ESR in order to determine **^.^**OH generation from freshly generated whole fume.

### Grouped filters

Grouped filter suspensions, were used for H_2_O_2_, lipid peroxidation, O_2 _consumption and DNA damage analysis. Suspensions were prepared by splitting the filters into three size groups. For this study the size groups were defined as ultrafine (0.01 - 0.056 μm), fine (0.10 - 1.0 μm) and coarse (1.8 - 18 μm) particles. The groups consisted of five filters each of which were placed in PBS and blended on ice into a fine slurry using a Tissue Tearor (Biospec Products Inc. Racine, WI). Pre- and post filter weights were used to prepare a final suspension of 1 mg/ml welding fume. The slurry was centrifuged, and the welding fume suspension was decanted from the filter pellet. A clean control suspension was also prepared at a ratio of 5 filters/1 ml PBS. Suspension not used immediately was aliquoted and frozen at -70°C.

### Lipid peroxidation

Lipid peroxidation of RAW 264.7 mouse peritoneal monocytes was measured by using a colormetric assay for malondialdehyde (LPO-586 Oxis International Inc. Portland, OR, USA). A reaction mixture contained various size groups (refer to filter methods) of welding particle samples [250 μg/ml], H_2_O_2 _(1 mM) and 1 × 10^7 ^cells in a total volume of 1.0 ml PBS (pH 7.4). A Fenton reaction, FeSO_4 _(1 mM), H_2_O_2 _(1 mM) and 1 × 10^7 ^cells, was also carried out as a positive control. The mixtures were exposed for 1 h in a shaking water bath at 37°C. The measurement of lipid peroxidation is based on the reaction of a chromogenic reagent with malonaldehyde at 45°C [53,54]. The absorbance of the supernate was measured at 586 nm.

### H_2_O_2 _production

H_2_O_2 _production was monitored using a Bioxytech quantitative hydrogen peroxide assay kit (H_2_O_2_-560, Oxis International Inc. Portland, OR, USA). Measurements were made on a system containing 5 × 10^6 ^RAW 264.7 mouse peritoneal monocytes/ml in pH 7.4 PBS and exposing them to the size groupings (refer to filter methods) of welding particle solution. Cells were exposed to welding particle solution [250 μg/ml], for 30 minutes in a 37°C incubator. Absorbance was monitored at a wavelength of 560 nm using a Spectra Max 250 multi-well plate reader (Molecular Devices, Sunnyvale, CA, USA).

### O_2 _consumption

Oxygen consumption measurements were carried out using a Gilson oxygraph (Gilson Medical Electronics, Middleton, WI). Measurements were made on a system containing 3 × 10^6 ^RAW 264.7 mouse peritoneal monocytes/mL and welding particle solution [500 μg/ml], in pH 7.4 phosphate buffer. The oxygraph was calibrated with media and equilibrated with known concentrations of oxygen.

### DNA damage, Comet assay

The Comet assay was performed using methods described in the Trevigen (Gaithersburg, Md., U.S.A.) assay kit. A typical reaction mixture contained welding particle solution [250 μg/ml], H_2_O_2 _(1 mM), and 1 × 10^7 ^RAW 264.7 mouse peritoneal monocytes cells brought to a total volume of 1.0 mL in PBS (pH 7.4). Briefly, (all steps performed in the dark or low light conditions) RAW264.7 mouse cells were exposed to the various sized welding fume and incubated in media at 37 °C for 1 h. The cells were washed (2×) with PBS and combined with LMAgarose, then pipetted onto a Comet slide. The slide was placed in a refrigerator for 30 min, immersed in lysis solution, chilled for 60 min, and then immersed in alkaline solution for 55 min. Slides were placed in a horizontal electrophoresis chamber for 40 min at 300 mA. Slides were washed and SYBR green stain was added to each. Slides were visualized using fluorescence microscopy, with an image capturing system (Olympus AX70 and sample PCI, Compix, Cranberry Township, PA). A minimum of 50 cells were scored for each sample at 400× magnification. The distance between the edge of the head and the end of the tail was measured using an automated image analysis system (Optimas 6.51, Media Cybernetics Inc., Silver Spring, Md) [55,56].

### Statistics

Data were expressed as mean ± standard error of the mean (SEM) (n = 4) for each group. One-way ANOVA test was performed using SigmaStat statistical software (Jandel Scientific, San Rafael, CA, USA) to compare the responses between treatments. Statistical significance was set at p < 0.05.

## Abbreviations

Defined in text

## Competing interests

The authors declare that they have no competing interests.

## Authors' contributions

SSL, BTC, DF and JMA contributed to the idea and design of the study. SSL, BTC and SGS carried out welding generation and particle collection. SSL and AJK carried out all electron spin resonance and toxicity measurements. DSB carried out EM analysis of particles. All authors read and approved the final manuscript.
